# Transcriptional Regulation of the Tumor Suppressor FHL2 by p53 in Human Kidney and Liver Cells

**DOI:** 10.1371/journal.pone.0099359

**Published:** 2014-08-14

**Authors:** Jiaying Xu, Junwei Zhou, Man-Shan Li, Chor-Fung Ng, Yuen-Keng Ng, Paul Bo-San Lai, Stephen Kwok-Wing Tsui

**Affiliations:** 1 School of Biomedical Sciences, The Chinese University of Hong Kong, Shatin, New Territories, Hong Kong; 2 Department of Surgery, The Chinese University of Hong Kong, Shatin, New Territories, Hong Kong; The University of Hong Kong, China

## Abstract

Four and a Half LIM protein 2 (FHL2) is a LIM domain only protein that is able to form various protein complexes and regulate gene transcription. Recent findings showed that FHL2 is a potential tumor suppressor gene that was down-regulated in hepatocellular carcinoma (HCC). Moreover, FHL2 can bind to and activate the TP53 promoter in hepatic cells. In this study, the activity of the two promoters of FHL2, 1a and 1b, were determined in the human embryonic kidney cell line HEK293 and the activation of these two promoters by p53 was investigated. Our results showed that the 1b promoter has a higher activity than the 1a promoter in HEK 293 cells but the 1a promoter is more responsive to the activation by p53 when compared with the 1b promoter. The regulation of FHL2 by p53 was further confirmed in liver cells by the overexpression of p53 in Hep3B cells and the knockdown of p53 in HepG2 cells. Combining promoter activity results of truncated mutants and predictions by bioinformatics tools, a putative p53 binding site was found in the exon 1a of FHL2 from +213 to +232. The binding between the p53 protein and the putative p53 binding site was then validated by the ChIP assay. Furthermore, the expression of FHL2 and TP53 were down-regulated in majority of HCC tumour samples (n = 41) and significantly correlated (*P* = 0.026). Finally, we found that the somatic mutation 747 (G→T), a hot spot mutation of the TP53 gene, is potentially associated with a higher expression of FHL2 in HCC tumour samples. Taken together, this is the first in-depth study about the transcriptional regulation of FHL2 by p53.

## Introduction

Four and a Half LIM protein 2 (FHL2) is known to function as an adaptor protein that is able to transactivate gene expression. Although it is predominantly expressed in the human heart tissue, its expression in a wide range of normal human tissues has been well documented [1]–[4]. FHL2 has been implicated in many forms of cancers in which its expression was found to be deregulated [5]. The function of FHL2 in cancers is particularly intriguing because FHL2 can function as an oncoprotein or as a tumor suppressor [6] in different type of cancers, including prostate cancer [7], liver cancer [8], [9], gastrointestinal cancers [10]–[12], breast cancer [13] and osteosarcoma [14]. The functional diversity of FHL2 could be contributed by its protein partners in different signaling pathways, e.g. the transforming growth factor β signaling pathway [8], [15] the NF-κB signaling [16], [17], the Wnt signaling [14] and estrogen receptor signaling [18].

Concerning the transcriptional regulation of FHL2, the human FHL2 gene expresses five transcript variants and all of them translate into the same FHL2 protein [19]. Among these variants, there are two alternative 5′ exons, namely exon 1a in transcript variant 4 and exon 1b in transcript variants 1, 2, 3 and 5. This implies that there are two promoters controlling the FHL2 expression. The 1b promoter, the upstream promoter of exon 1b, has been previously characterized [19].

On the other hand, the p53 protein is known to be a tumour suppressor protein that is crucial in the cell cycle regulation. Mutations or deletions of the TP53 gene can be fatal and are found in more than 50 percent of human tumours. Although there is no direct interaction between FHL2 and p53 [20], [21], earlier studies showed that there were significant differences in FHL2 mRNA levels in myoblasts and osteoblasts expressing wild-type TP53 or mutant TP53 [22], [23]. Using a temperature-sensitive TP53 mutant or γ-irradiation to activate TP53 expression in RD cells or human peripheral blood lymphocytes, the FHL2 mRNA level was elevated [23], [24]. In addition, it was shown that FHL2 enhances p53-dependent transcriptional activity through a direct association with HIPK2 [21]. A later studies in gliomas showed that the overexpression of FHL2 increased the tumourigenicity of glioblastoma cells in nude mice and decreased the mRNA levels of TP53 and its downstream pro-apoptotic genes [25]. Further investigation in *Apo-FHL2* mice demonstrated that FHL2 can bind and activate the TP53 promoter, and inactivation of TP53 was sufficient to block FHL2-induced apoptosis in hepatic cells [9]. Last but not least, it has been shown that FHL2 can compete with p53 for binding the transcription factor E4F1 in human osteosarcoma U2OS cells and inhibit the anti-proliferative functions of E4F1 by inhibiting its transcription repressor function and p53 binding activity of E4F1 in the cells [26].

Here, we report the characterization of the FHL2 1a promoter and the differential activity of 1a and 1b promoter in HEK293 cells. Moreover, we studied the transcriptional regulation of FHL2 by p53 mediated by 1a and 1b promoters. Our results showed that the 1b promoter is more active than the 1a promoter in HEK 293 cells but the 1a promoter is more responsive to the activation by p53 when compared with the 1b promoter. The same differential action could be also observed in liver Hep3B cells. Furthermore, the expression of FHL2 and TP53 were down-regulated in majority of HCC tumour samples (n = 41) and significantly correlated (*P* = 0.026). Finally, we have identified a putative p53 binding site in the 1a promoter and a hot spot mutation of the TP53 gene that is potentially associated with a higher expression of FHL2 in HCC tumour samples. Taken together, this is the first in-depth study about the transcriptional regulation of FHL2 by p53.

## Materials and Methods

### Ethics Statement

The study was approved by the Joint CUHK-NTEC Clinical Research Ethical Committee at the Prince of Wales Hospital in Hong Kong. Written informed consent was obtained from all the studied subjects for sample collection and subsequent analysis.

### Cell lines and cell culture method

HEK 293 (human embryonic kidney cells), as well as HepG2 and Hep3B (human liver cancer cells) were purchased from ATCC and maintained in Dulbecco’s modified Eagle medium (DMEM) or Roswell Park Memorial Institute (RPMI) - 1640 medium (Invitrogen, Carlsbad, CA, USA). The medium was supplemented with 10% fetal bovine serum (FBS) and 1% antibiotic penicillin/streptomycin. Cells were grown in 75 cm^2^ culture flask and were maintained at 37°C, 5% CO_2_ humidified incubator. They were sub-cultured using 0.25% trypsin-EDTA every 2–3 days before reaching about 90% confluence. Both cell lines were adherent cells.

### Quantitative real-time PCR

Real-time PCR analysis of gene expression was performed with ViiA 7 Real-Time PCR System (Applied Biosystems). Real-time PCR was performed in 96-well optical plates and the real-time PCR mixtures (final volume 10 µl) consists of 2 µl cDNA (diluted 1∶10 in nuclease-free water), 5 µl Power SYBR Green PCR Master Mix (2×) (Applied Biosystems) and 125 nM forward and reverse primers. All samples were analyzed in triplicates and no-template controls were included for every experiment. The PCR cycling conditions included an initial step of 50°C for 2 minutes and initial denaturation at 95°C for 10 minutes, followed by 40 cycles of amplification at 95°C for 15 seconds and 60°C for 1 minute. Dissociation analysis was performed at the end of each run to check the specificity of PCR reaction. The quality and quantity of PCR product was also checked by gel electrophoresis. The comparative C_T_ method (2^−ΔΔCT^) was used to calculate the relative amounts of each mRNA between samples, normalized to the endogenous housekeeping gene β-actin. C_T_ value represents average of the triplicate C_T_ values.

### Western blot analysis

An equal amount of protein (50 µg) was mixed with 4× loading buffer, which was boiled for 5 minutes and subjected to SDS polyacrylamide gel electrophoresis. After that, the proteins were transferred onto a polyvinylidene difluoride membrane (Immobilon P, Millipore) at 15 V for 70 minutes using a semi-dry transfer system *(*Bio-Rad*)*. The membrane was blocked with 5% non-fat milk in TBST for 30 minutes, probed with primary antibody overnight at 4°C under gentle agitation, followed by wash for three times in TBST and incubation with secondary antibody (1∶5000) for 1 hour at room temperature. The membrane was washed three times in TBST and then developed by Western Lightning Chemiluminescence Reagent Plus (Perkin Elmer Life Sciences) and autographed on X-ray film using a medical x-ray processor (model 102, Kodak). The following primary antibodies were used in the Western blot analysis: mouse monoclonal anti-FHL2 (1∶1000), mouse monoclonal anti-TP53 (1∶1000) and mouse monoclonal anti-β-actin (1∶5000; all from Santa Cruz Biotechnology). Anti β-actin was used for normalization.

### Cloning of FHL2 1a promoter and deletion mutants

The 5′ flanking region of exon 1a of the human FHL2 gene was amplified by PCR from human genomic DNA using the primers P–2139luc-F and P+223luc-R and cloned into the *Kpn*I/*Nhe*I sites of pGL4.10 empty luciferase vector (Promega) to generate the FHL2 1a promoter luciferase reporter construct. For the construction of deletion mutants, different lengths of the 5′-flanking region were amplified from the reporter plasmid by PCR using the same reverse primer and the forward primers, followed by subcloning into the same restriction enzyme sites of pGL4.10. All clones were verified by PCR and DNA sequencing.

### Cloning of the TP53 expression vector

The complete coding region of human TP53 (Accession number: M14695.1) was amplified by PCR using the primers TP53-mRNA-F and TP53-mRNA-R. The PCR fragment (1207 bp) was recovered from the agarose gel, and cloned into *Hind*III and *Bam*HI sites of pcDNA3.1(+) empty vector (Invitrogen) to obtain the pcDNA3.1-TP53 expression vector. The identity of the clone was confirmed by PCR and DNA sequencing.

### Transient transfection and dual luciferase reporter assay

HEK 293 cells were plated at 1×10^5^ cells in 0.5 ml complete medium per well in 24-well plates at 70–80% confluence. After 24 hours, cells were transfected with 0.4 µg of deletion constructs and 40 ng of pSV-Ruc, the *Renilla* luciferase vector per well using Lipofectamine Transfection Reagent (Invitrogen). A ratio of 1 µg DNA to 2.5 µl Lipofectamine reagent ratio was used. After 48 h of transfection, dual luciferase assay (Promega) was performed to study the promoter activity following the manufacturer’s protocol. The promoter activity was expressed as arbitrary units normalized to *Renilla* luciferase activity to control the transfection efficiency for each well. For TP53 co-transfection, 0.2 µg TP53 expression vector or pcDNA3.1 empty vector with 0.4 µg of deletion constructs and 40 ng of pSV-Ruc were used and measured with the same method mentioned above.

### Promoter analysis and transcription factor binding site prediction

Transcription factor binding sites in the full-length FHL2 1a (–2139 to +375) and 1b (–2268 to +397) promoters were predicted by four commonly used computational programs - PATCH v1.0 public [27], PROMO v3.0.2 [28], [29], MatInspector v8.06 professional [30] and JASPAR [31]. Only binding sites predicted by more than one program were further subjected to the multiple genomic alignments. To identify conserved nucleotide sequences that may be functionally important in different species, Ensemble Genome Browser (http://www.ensembl.org/) was used for multiple genomic alignment of FHL2 in 12 different eutherian mammals.

### Mutagenesis of predicted p53 binding site

The core sequence (CAATGTCCAGACGTGCCTTA) of predicted TP53 binding site within the FHL2 1a promoter p+131 was randomly mutated (TGCCTCAACT TAAGAAAGGC) by direct annealing of the primers M-TP53-F and M-TP53- R. Briefly, annealing was performed in 100 µl mixtures containing 1 mM of each primer and 1x annealing buffer. The tube was kept in a heat block at 95°C for 15 minutes and then naturally cooled down to room temperature for 60 minutes. End repair was carried out in 50 µl reaction mixtures containing 10 µl of annealing product, 1x PCR Buffer, 100 mM of MgCl_2_, 10 mM of dNTPs and 0.5 U *Taq* DNA polymerase. After incubation at room temperature for 2 hours, blunt-ended DNA product (146 bp) was recovered from the agarose gel, and cloned into the *Kpn*I/*Nhe*I sites of pGL4.10 empty luciferase vector (Promega) to generate the mutant TP53 binding site in FHL2 1a p+131 promoter. The identity of the clone was confirmed by DNA sequencing.

### Overexpression of p53 in Hep3B cells

Hep3B cell lines were seeded in 6-well plate at the density of 2×10^5^ cells per well. When the cells confluence reached around 80%, plasmids pcDNA3.1 and pcDNA3.1-p53 were transfected into the cells using Lipofectamine 2000 (Invitrogen, Carlsbad, CA, USA) according to the manufacturer’s procedures, respectively. Total RNA was isolated 24 hours post-transfection with Trizol reagent (Invitrogen, Carlsbad, CA, USA) following the manufacturer’s instruction. cDNA was synthesized by 1 µg RNA with QuantiTect Rev Transcription Kit (Qiagen, GmbH. Hilden, Germany). Real-time PCR was performed using 2× Power SYBR Green PCR Master Mix (Applied Biosystems, Foster City, CA, USA) with ABI 7900 Fast Real-time PCR system. The PCR conditions was 95°C for 10 min followed by 40 cycles of 95°C for 15 s and 60°C for 1 min. Specific primers for FHL2-1a (FHL2-la-F: 5′-GGG AGC TGA GGC TTC TTT AA-3′ and FHL2-la-R: 5′-CCT TGT CAG TGG CGC TAT AA-3′) and TP53 (TP53-F: 5′-TCC TCA GCA TCT TAT CCG AG-3′ and TP53-R: 5′- CAC CAC CAC ACT ATG TCG AA-3′) were used. The house-keeping gene β-actin (*ACTB*) was used as internal control. Comparative C_T_ method (2^−ΔΔCT^) was used to calculate the relative expression level of the mRNA normalized to *ACTB.* Total protein was extracted at 48 hours post-transfection by using the RIPA (Cell Signaling Technology, Danvers, USA). The p53 antibody (Santa Cruta Biotechnology, Texas, USA) was used to detect p53 protein. The signal of p53 protein was visualized on X-ray film after treating with Western Lightning Chemiluminescence Reagent Plus (Perkin Elmer Life Science, Massachusetts, USA).

### Knockdown of p53 in HepG2 cells with siRNA

Two Small interfering RNAs (siRNAs) including siRNA against human p53 (p53-siRNA, sc-29435) and control scrambled siRNA (scRNA, sc-37007) were purchased from Santa Cruz Biotechnology (Santa Cruz). HepG2 cells were seeded in 6-well plate at a density of 2×10^5^ cells per well. When confluence reached around 70%, cells were transfected with p53-siRNA and control siRNA, respectively, by using Lipofectamine RNAiMAX Reagent according to the manufacturer’s protocol. Total RNA and protein were harvested at 24 hours and 48 hours post-transfection. The expression level of p53 and FHL2 were determined by real-time PCR and Western blotting.

### Chromatin immunoprecipitation (ChIP) assay

Hep3B cells overexpressed with p53 and HepG2 cells with the knockdown of p53 were used for ChIP analysis at 48 hours post-transfection. ChIP assay was performed by using the Champion Chip One-Day Kit (SABiosciences Corporation Company, MolBio, GmbH, Germany) following the manufacture’s instruction. Mouse monoclonal antibody against p53 (Santa Cruta Biotechnology, Texas, USA) was used for DNA fragment enrichment. The immunoprecipitated chromatin was analyzed in triplicate by real-time PCR using primers specific to the putative p53 binding site (FHL2-Chip-F: 5′-GGC AGG ACA AAC GAG GAC ATG GC-3′ and FHL2-Chip-R: 5′-GGC CCC AAC CTT CTG TGC AGC TG-3′).

### Clinical samples and sample selection criteria

Tumourous HCC tissues and adjacent non-tumourous tissues (at least 1-cm distance away from the tumour edge) were collected from patients who underwent surgical resection at the Prince of Wales Hospital, Hong Kong between 1998 and 2005. Written consent was obtained from the patients prior to tissue harvesting and the study protocol was approved by the ethics committee of The Chinese University of Hong Kong. The tissues were immediately snap-frozen in liquid nitrogen and stored at −80°C until processed for RNA extraction. Human tissue samples were randomly selected from our tissue collection. Samples with definitive demographic, clinicopathological and follow-up records were used for our analyses. All the selected tissues had the following characteristics:- (1) collected before any treatment; (2) with/without HBV/HCV infection; (3) age ≥20 years old; (4) primary HCC; and (5) non-necrotic. The characteristics of patients are listed in Table S1 in File S1.

### Sequencing of TP53 cDNA in HCC samples

Primers (TP53-CDS-F/R) were designed to amplify a full-length (1205 bp) product that covers the entire coding region of TP53 gene in HCC patient samples. The cDNA prepared from the tumourous tissues and adjacent non-tumourous tissues of HCC patients were used as template for PCR. Afterwards, 5 µl of PCR products were checked by 1% agarose gel electrophoresis and the remaining PCR products were sequenced directly using the same primers used for amplification. For those cDNA samples that cannot be amplified by full-length primers, three pairs of primers which divided the coding region of TP53 into three sections were used for further amplification and sequencing.

### Statistical analysis

The results shown represent mean ± SEM from triplicate samples unless otherwise specified. The promoter activities were compared by unpaired t-test. *P*<0.05 is considered to be statistically significant. Statistical analyses were performed by using GraphPad Prism 5 software (GraphPad Software Inc.). For the study of the correlation between the expression of FHL2 and TP53, comparative C_T_ method (2^−ΔΔCT^) was used to calculate the relative level of the mRNA normalized to *ACTB* and correlation analysis was performed using SPSS (version 16.0).

## Results

### Deletion mutation analysis of FHL2 1a and 1b promoters

**Figure 1:**
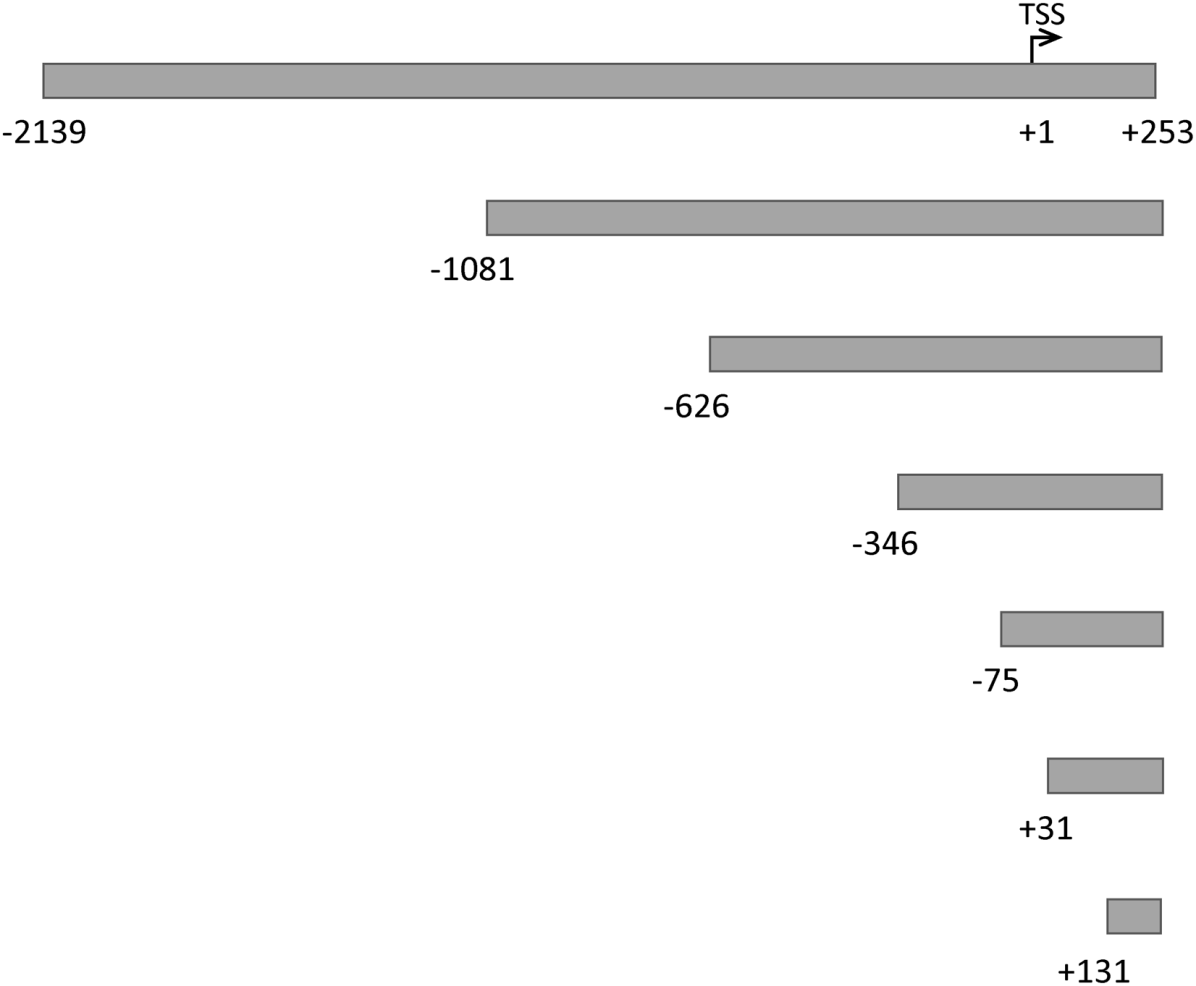
Schematic diagram of truncated 5′- flanking sequences of FHL2 exon 1a. These truncations were fused to the luciferase reporter gene in the pGL4.10-basic vector. Numbers indicate positions relative to the transcription start site (TSS, +1).

**Figure 2:**
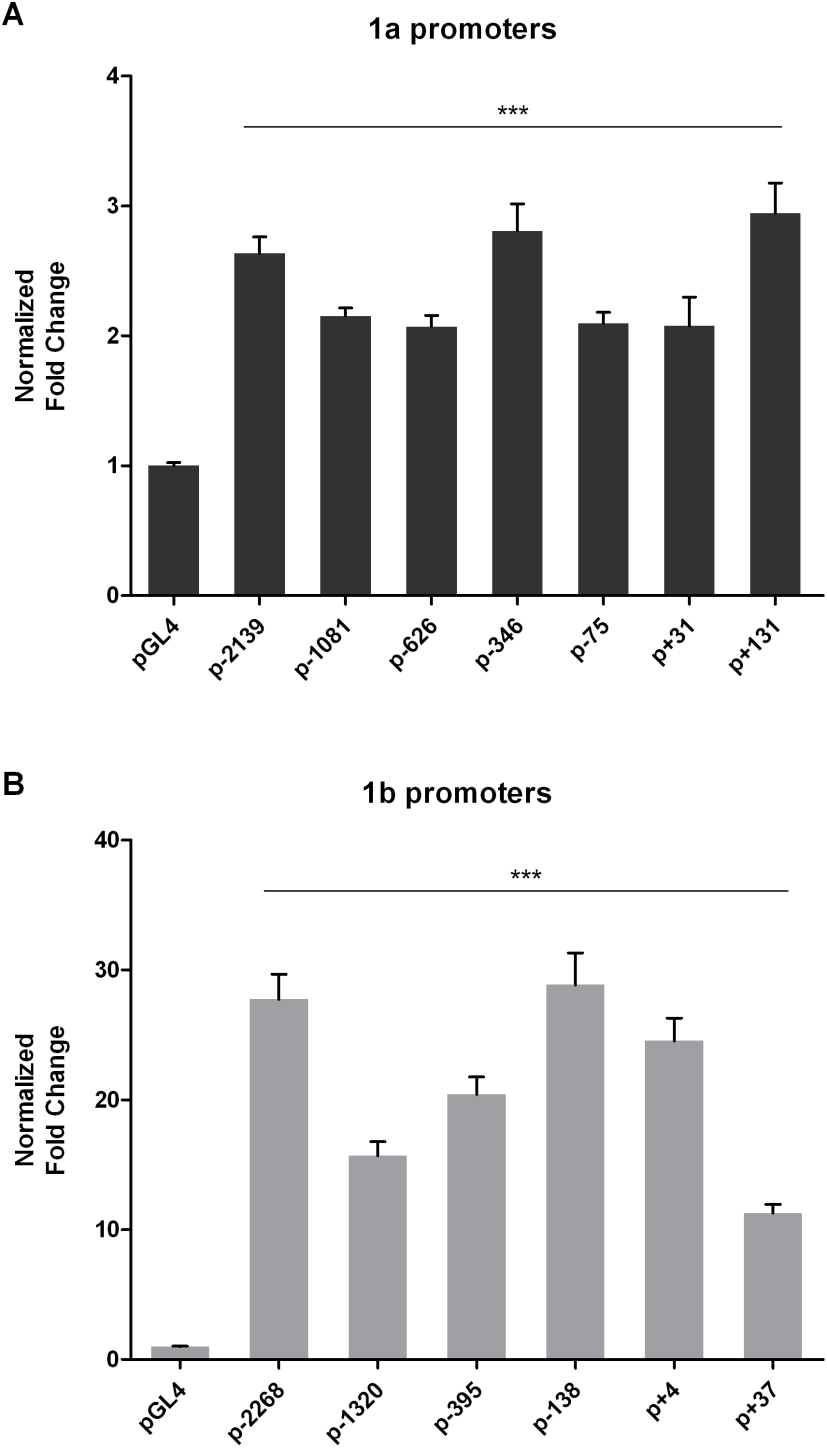
Activities of truncated FHL2 1a (A) and 1b (B) promoters were determined in HEK293 cells. Data were normalized by pGL4 and calculated as mean ± SEM from three independent experiments, each done in triplicates. Triple asterisks (***) indicates *P*<0.001 versus control group (pGL4) by unpaired t-test.

Exon 1a, which lies 40 kb upstream of exon 1b, is the first exon of FHL2 gene. Among the five transcript variants of FHL2, exon 1a is only found in transcript variant 4, which followed by the coding sequence from exon 4 to 8. So far, there is little information about the 5′-flanking region of the exon 1a. To identify the activity of FHL2 1a promoter, a series of deletion mutants was constructed by inserting truncated promoters of different lengths into the pGL4.10-basic vector for luciferase activity analysis. A schematic diagram of these constructs is shown in Figure 1. Both truncated 1a and 1b promoters were transfected into HEK 293 cells and then luciferase activities were determined. Interestingly, all truncated 1a promoters showed a similar and significant increase in activities by 2 to 3 folds when compared with pGL4.10-basic vector (Figure 2A). In contrast, the truncation mutants of the 1b promoters showed a gradual increase in activities from −1320 to −138 and then the activities decreased in subsequent mutants. In the mutant with a deletion up to −138, the highest activity of nearly 30 folds compared with the vector control was observed (Figure 2B). In summary, the FHL2 1b promoter has a higher activity than the 1a promoter in HEK 293 cells.

### TP53 overexpression activates FHL2 at the transcriptional level

**Figure 3:**
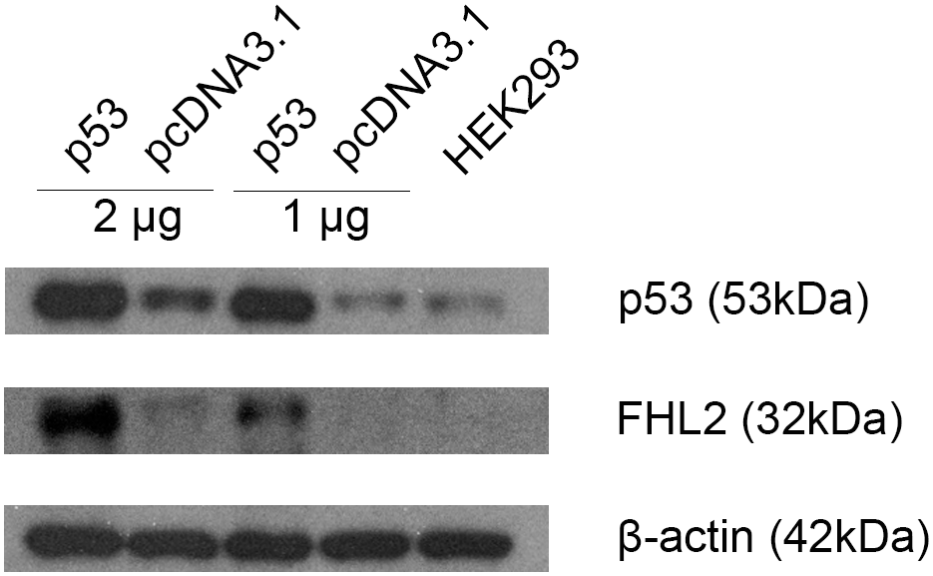
Protein expression of TP53 and FHL2 were detected by western blotting after transfection of 1 or 2 µg TP53 expression plasmid in HEK 293 cells for 48 h. pcDNA3.1 served as empty vector control of TP53 and the untreated HEK 293 cells lysate showed the endogenous levels of p53 and FHL2. β-actin was used as internal control.

It was previously reported that FHL2 is a p53-responsive gene [23]. In this study, we retrospectively analyzed expression patterns of FHL2 when overexpressing TP53 in different concentrations. Briefly, 1 µg and 2 µg of TP53 expression plasmid or pcDNA3.1 vector plasmid were individually transfected into HEK 293 cells. Western blotting results in Figure 3 showed the successful over-expression of p53 protein after 48 h of transfection. Endogenous p53 can also be detected in HEK 293 cells, but its level was much less than those transfected with the TP53 expression plasmid. The increase of FHL2 expression in a p53-dependent manner was observed.

**Figure 4:**
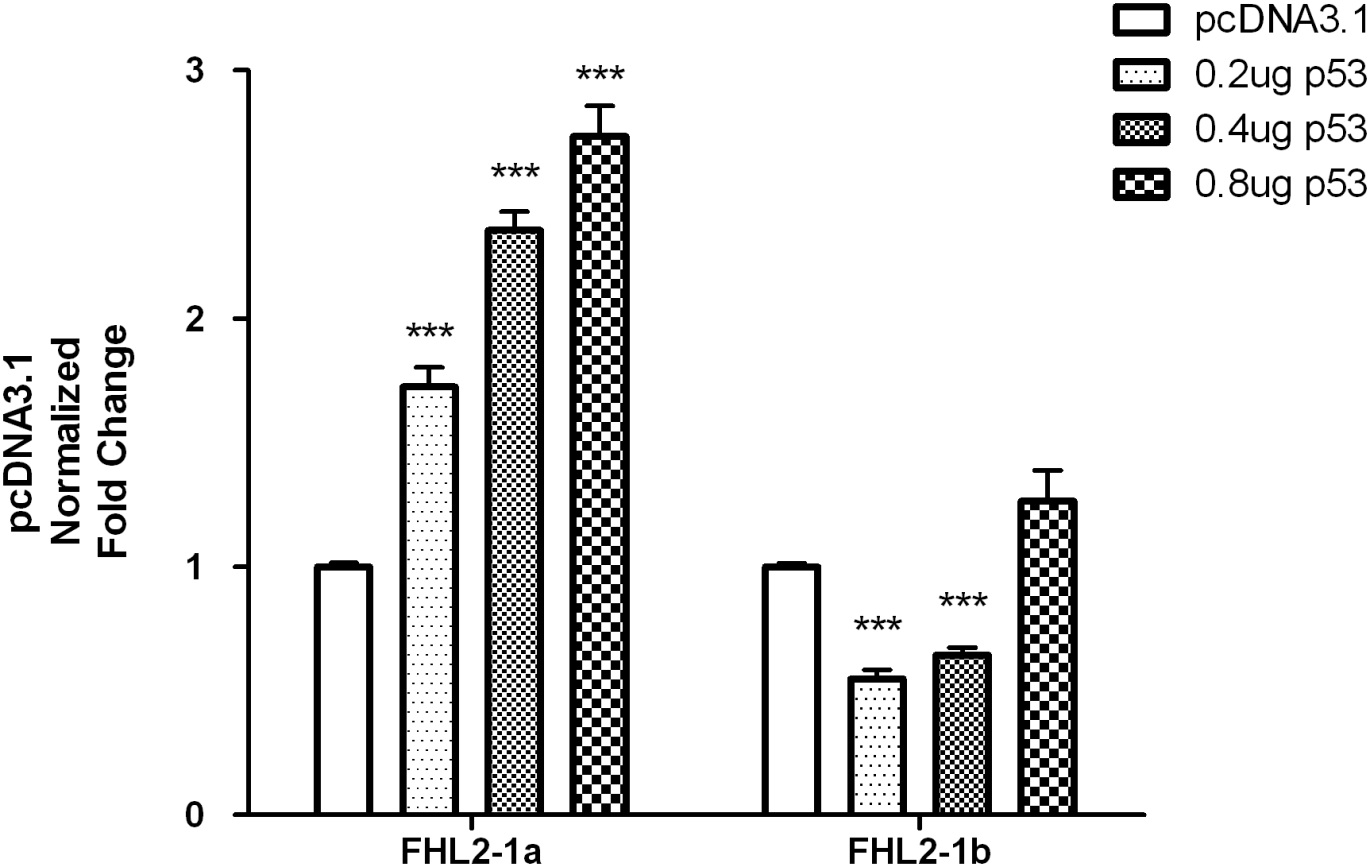
Activities of FHL2 1a and 1b 2k-promoters co-transfected with different concentrations of TP53 expression plasmid in HEK293 cells. Data obtained from three independent experiments, each done in triplicates. Data were normalized by pGL4 and pcDNA3.1 and presented as mean ± SEM. Triple asterisks (***) indicates *P*<0.001, double asterisks (**) indicates *P*<0.01 and single asterisk (*) indicates *P*<0.05 by unpaired t-test.

**Figure 5:**
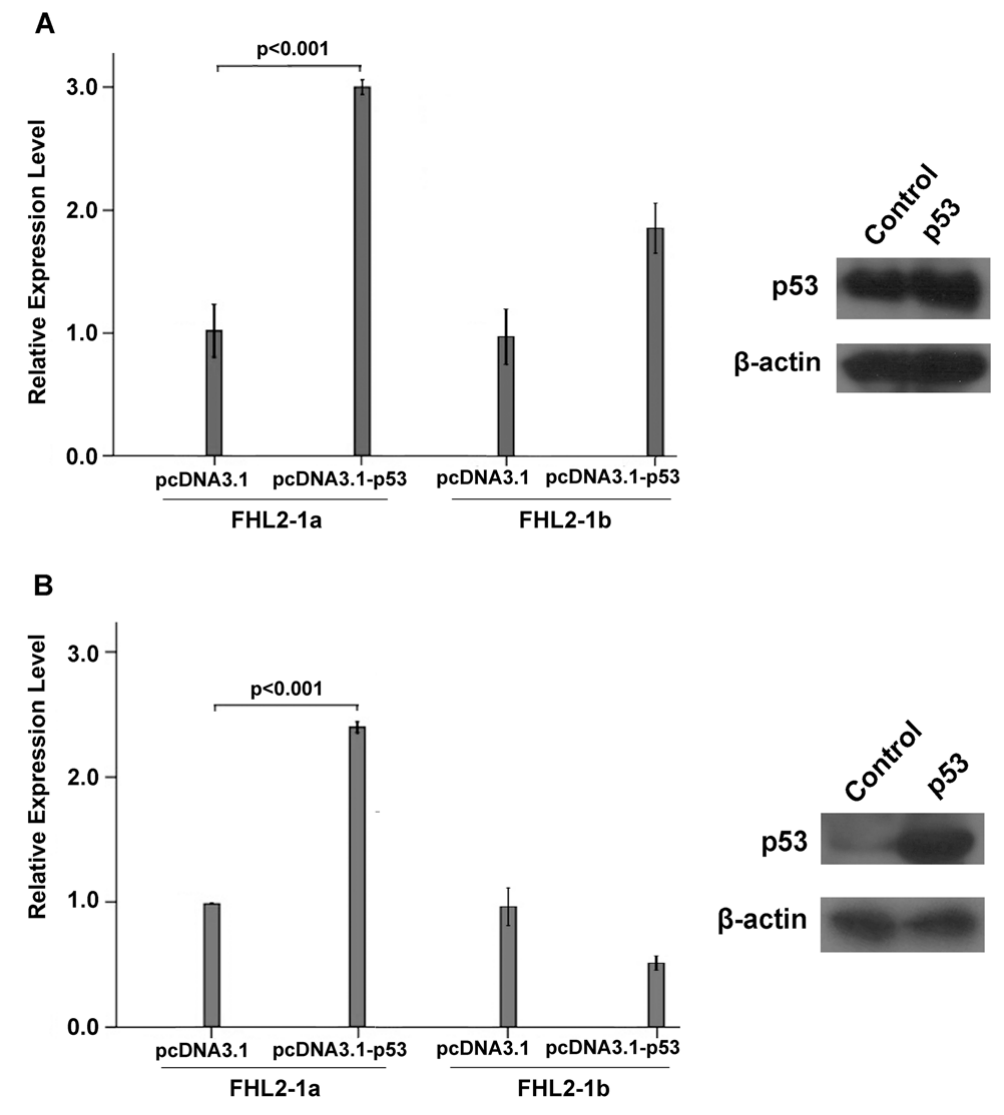
Relative expression levels of FHL2-1a and FHL2-1b upon over-expression of TP53 in HEK293 and Hep3B cell lines. TP53 expression plasmid was transiently transfected in HEK293 and Hep3B cells using empty pcDNA3.1 vector as control. Western blotting analysis indicated the successful over-expression of TP53 in both HEK293 and Hep3B cells. The mRNA levels of FHL2-1a and FHL2-1b were determined by real-time PCR. (A) The mRNA levels of FHL2-1a and FHL2-1b in HEK293 cells transfected with pcDNA3.1-TP53 and empty pcDNA3.1 vector. (B) The mRNA levels of FHL2-1a and FHL2-1b in Hep3B cells transfected with pcDNA3.1-TP53 and empty pcDNA3.1 vector.

To investigate the differential regulation of the two promoters by TP53 overexpression, both 2 kb-promoters of 1a (p-2139) and 1b (p-2268) were used for co-transfection with different amounts of TP53 expression vector. Results showed that the 1a promoter activity was significantly rose in a dose-dependent manner (Figure 4) while there was only slight fluctuation for the 1b promoter activity upon the overexpression of TP53. Moreover, the specific effect of p53 on the FHL2 1a transcript was further confirmed by real-time PCR using transcript-specific primers (Figure 5A). To see whether similar effect could be observed in liver cells, Hep3B cells were transfected with the expression plasmid of TP53. We found that FHL2 1a transcript was consistently and specifically up-regulated (Figure 5B).

**Figure 6:**
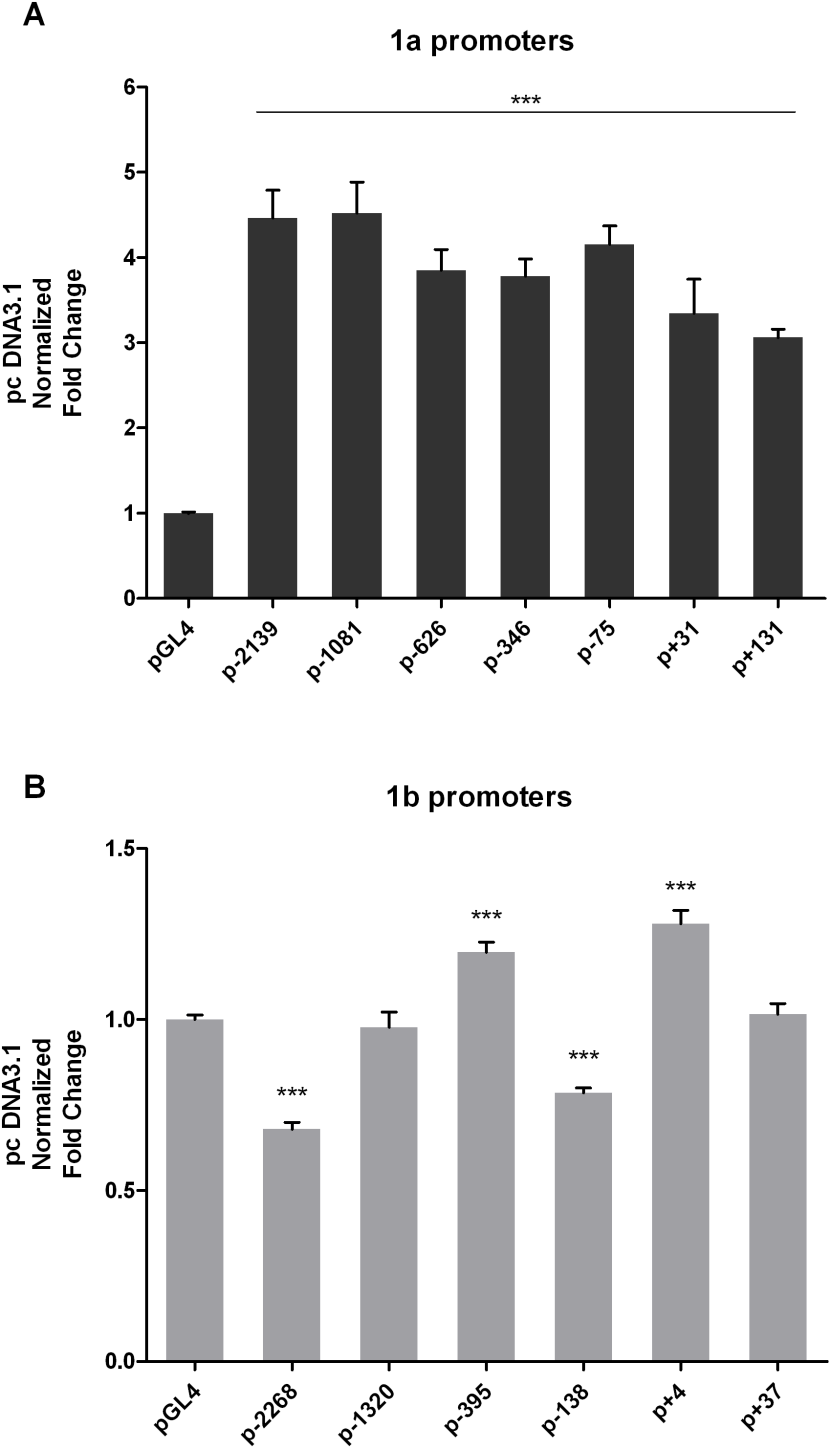
Activities of truncated FHL2 1a (A) and 1b (B) promoters co-transfected with p53 in HEK293 cells. Data obtained from three independent experiments, each done in triplicates. (**A** and **B**) Data were normalized by pcDNA3.1 and presented as mean ± SEM. Triple asterisks (***) indicates *P*<0.001 versus control group by unpaired t-test.

To further evaluate the effects of p53 on FHL2, activities of all truncated mutants of FHL2 1a and 1b promoters during the TP53 overexpression were determined (Figure 6). To our surprise, under the overexpression induced by 0.2 µg TP53 expression plasmid, all truncated mutants of 1a promoter have elevated activities from 3 to 5 fold compared with the control vector (Figure 6A). The elevations gradually dropped when the truncated mutants became shorter. However, the truncated mutants 1b promoters were not very sensitive to the TP53 overexpression and none of them showed a fold change greater than two (Figure 6B). Taken together, we conclude that the remarkable up-regulation of FHL2 in HEK293 cells induced by p53 is probably mediated by the FHL2 1a promoter.

### Bioinformatic analysis of p53 binding sites on the FHL2 promoter

To identify the possible p53 binding sites in the promoter regions of FHL2 gene, the full-length DNA sequences of the 1a (–2139 to +375) and 1b (–2268 to +397) promoters were analyzed by four commonly used computational TFBS prediction programs. They are: i) PATCH v1.0 Public, ii) PROMO v3.0.2, iii) MatInspector v8.06 Professional and iv) JASPAR. Since different databases and matrix profiles were used by these programs, the predicted binding site would be more reliable if it was commonly predicted by different programs. The predicted p53 binding sites were further analyzed by multiple sequence alignments using the Ensemble Genome Browser, where the conserved regions among different species were highlighted. In Figure S1 in File S1, the putative p53 binding sites within the evolutionary conserved region were shown. Four of them were found in the 1a promoter (–1871 to –1838, −1013 to −991, +88 to +129, +208 to +242) while the other two sites were found in the 1b promoter (–1118 to –1096, −206 to −170). Since previous results have shown that the TP53 overexpression is able to increase the promoter activity of even the shortest 1a promoter (p+131) by 3-fold, it is very probable that the p53 binding site is located within the exon 1a from +131 to +253. Thus, the predicted p53 binding site at the region of +208 to +242 was chosen for validation by site-directed mutagenesis.

### Mutagenesis of a predicted p53 binding site in FHL2 1a promoter

**Figure 7:**
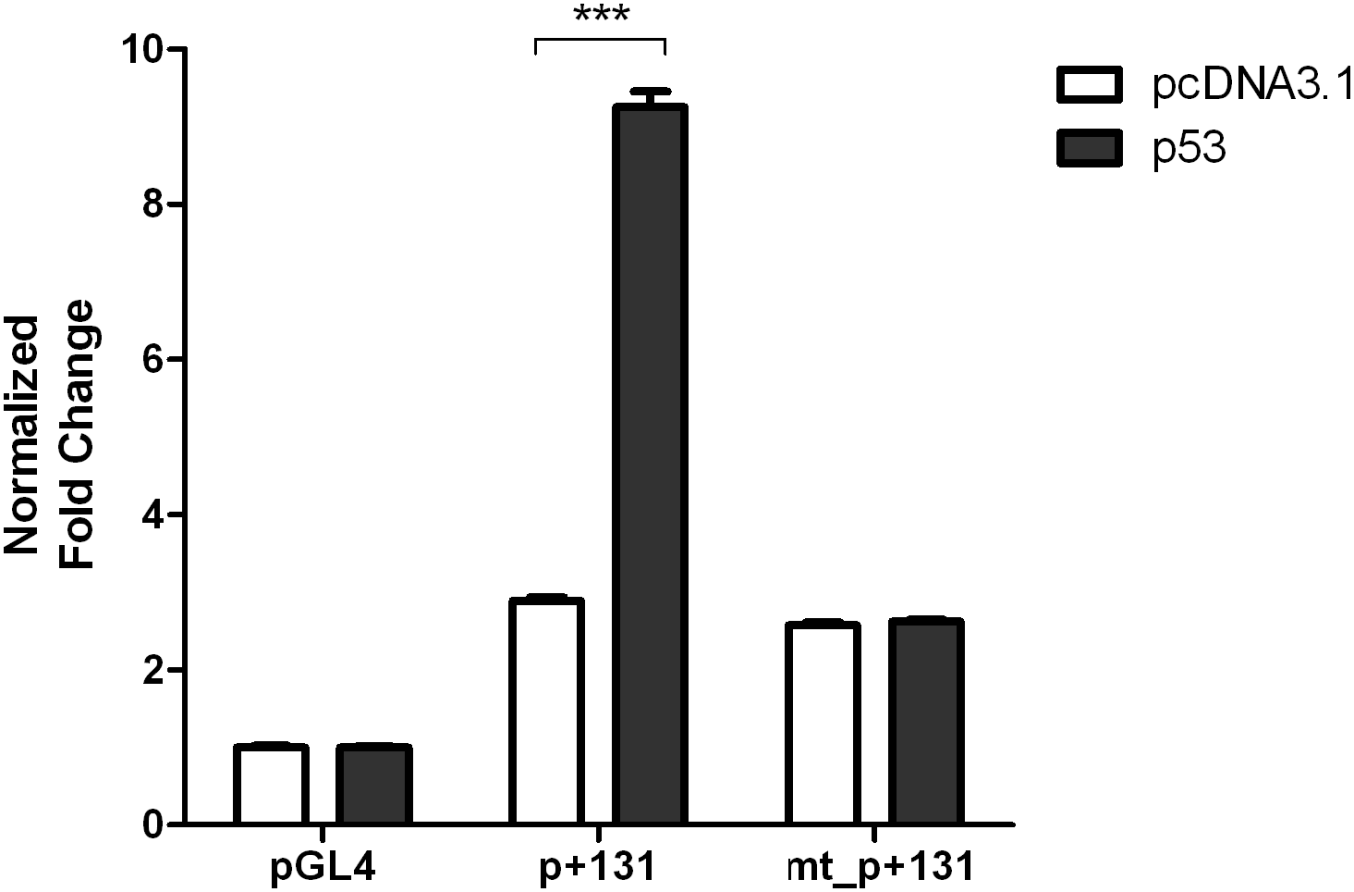
Activities of mutated FHL2 1a promoter p+131 co-transfected with TP53 expression plasmid in HEK293 cells. Data obtained from experiment in triplicates. Data were normalized by pGL4 and presented as mean ± SEM. Triple asterisks (***) indicates *P*<0.001 versus control group by unpaired t-test.

According to the p53 weight matrix of the prediction programs, a core sequence (CAATGTCCAGACGTGCCTTA) within the predicted p53 binding site at +213 to +232 was identified. We further mutated the core sequence in FHL2 1a promoter p+131 by replacing the core sequence with a random sequence, resulting in a mutant promoter named mt_p+131. Using the similar methods as previous described, the wild-type p+131 and mutant mt_p+131 promoters were co-transfected with the TP53 expression plasmid into HEK 293 cells. The luciferase activity results showed that the p53 mediated activation in promoter p+131 was completely abolished in the mutated promoter mt_p+131 (Figure 7). In conclusion, a putative p53 binding site contributing to the transcriptional activation of FHL2 is found in the exon 1a of the FHL2 from +213 to +232.

### Regulation of FHL2 by p53 in liver cells

**Figure 8:**
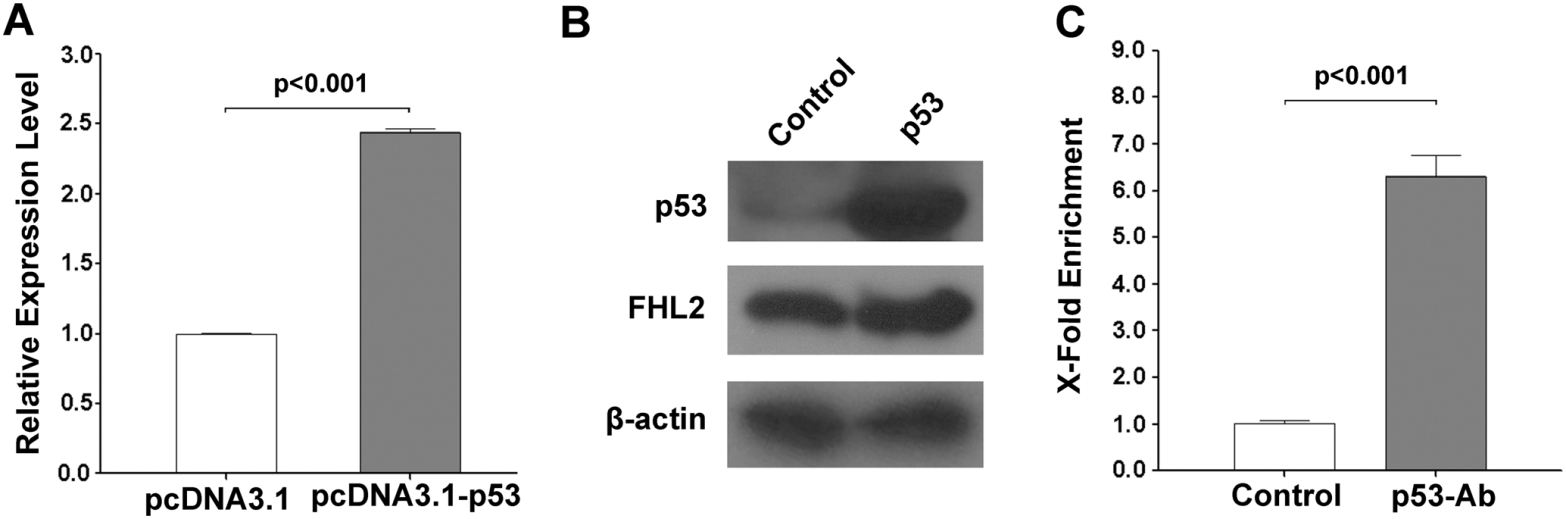
Overexpression of p53 could up-regulate the FHL2 expression in Hep3B cells. (**A**) Relative expression levels of FHL2-1a in Hep3B cells transfected with pcDNA3.1-p53 using pcDNA3.1 empty vector as control. (**B**) Protein expression of p53 and FHL2 were detected by western blotting after transfection with pcDNA3.1 and pcDNA3.1-p53. (**C**) ChIP analysis of p53-enriched DNA fragment for Hep3B cells overexpressed with p53. DNA fragment enriched in the ChIP assay were tested by qPCR. The p53 antibody used for enrichment was indicated in the x axis. Y axis represents fold enrichment of immunoprecipitated DNA (gray bars) against the control (empty bars).

**Figure 9:**
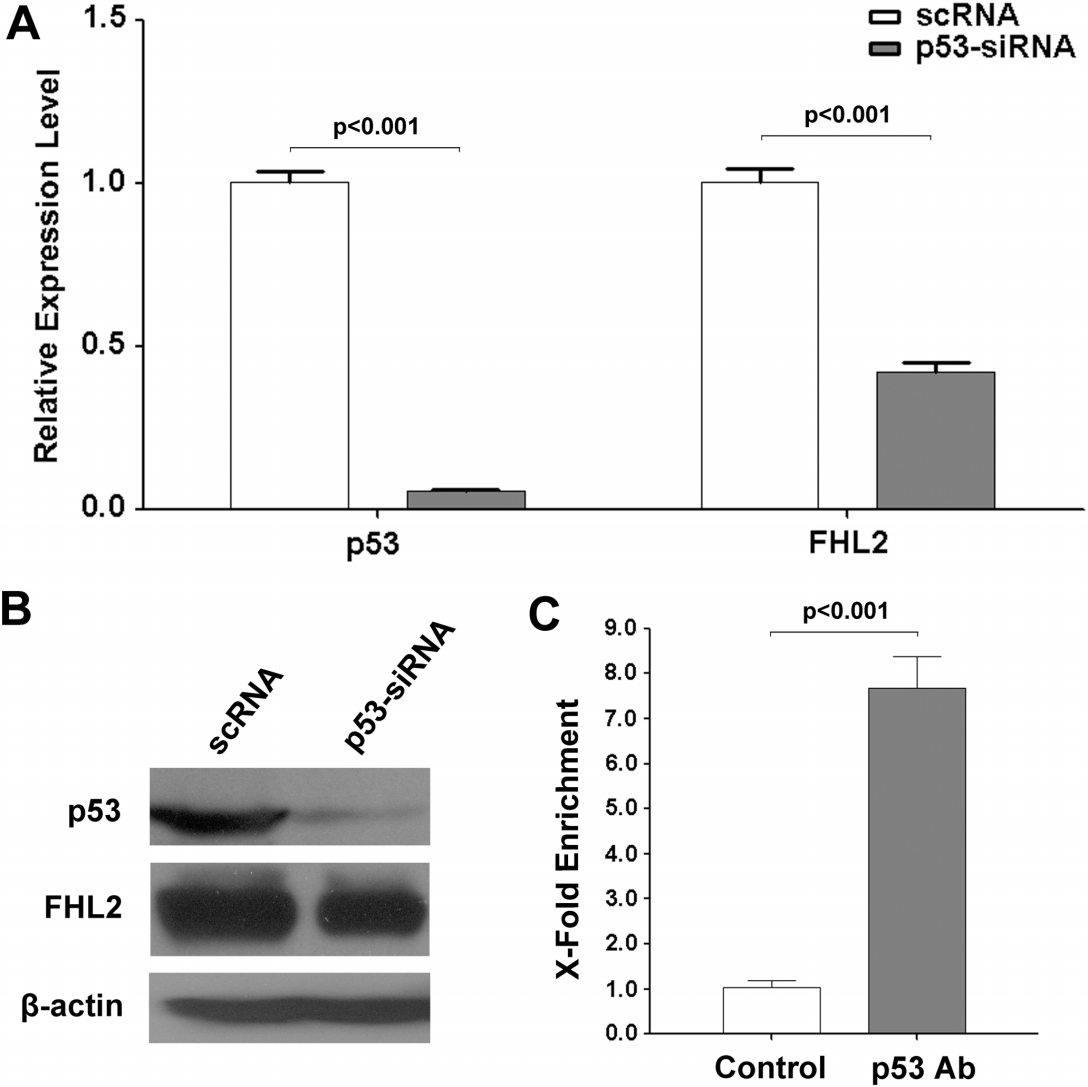
Knock-down of p53 could decrease the FHL2 expression in HepG2 cells. (**A**) Relative expression levels of p53 and FHL2-1a in HepG2 cells treated with p53-siRNA with scRNA as control. (**B**) Protein expression of p53 and FHL2 were detected by western blotting after siRNA treatment. (**C**) ChIP analysis of p53-enriched DNA fragment for HepG2 cells. DNA fragment enriched in the ChIP assay were tested by qPCR. The p53 antibody used for enrichment was indicated in the x axis. Y axis represents fold enrichment of immunoprecipitated DNA (gray bars) against the control (empty bars).

To demonstrate the regulation of FHL2 by p53 in liver cells, we overexpressed p53 in Hep3B cells, which has a relatively low expression level of p53. As shown in Figure 8, the expression of FHL2 was significantly increased in Hep3B cells upon overexpression of p53. Results of ChIP assay showed that the over-expressed p53 protein could bind to the putative p53 binding site at the exon 1a of the FHL2 promoter. To further confirm these results, we had knockdown p53 in HepG2 cells, a liver cell line that has a relatively high expression level of p53. We found that the expression of FHL2 was significantly decreased in HepG2 cells upon knockdown of p53 (Figure 9). Results of ChIP assay also indicated that the endogenous p53 could bind to the putative p53 binding site.

### Mutation analysis of the TP53 gene in HCC clinical samples

**Figure 10:**
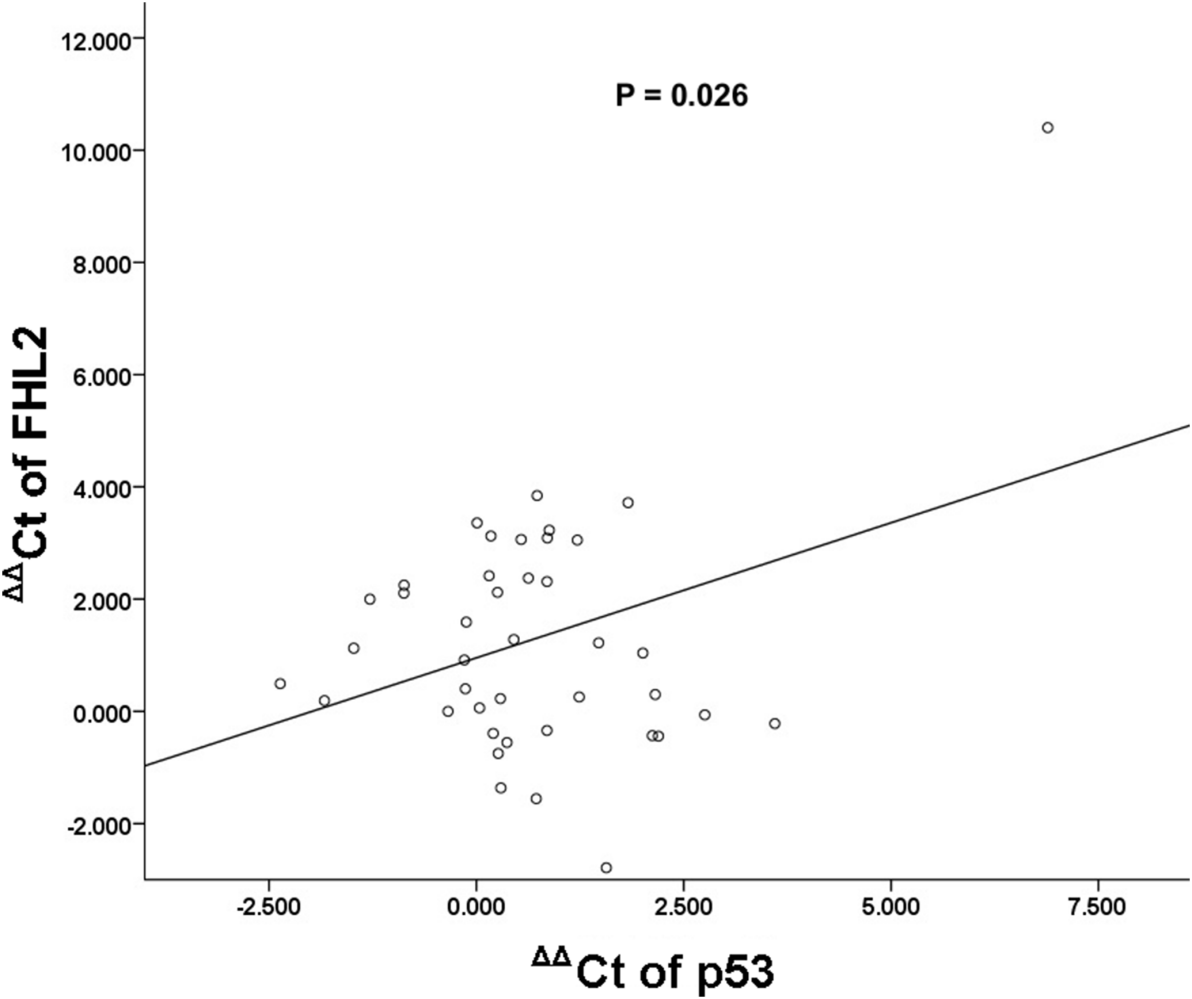
The correlation between the expression levels of FHL2 and TP53. The real-time PCR results were analysed using SPSS (version 16.0).

The gene expression levels of FHL2 and TP53 in samples of human HCC and adjacent normal tissues were determined by real-time PCR. Among these 41 samples, 38 samples were FHL2 down-regulated (93%) and 36 samples were TP53 down-regulated (89%). We found that there is a statistically significant correlation (*P* = 0.026) between the expression of FHL2 and TP53 in HCC samples when compared with their non-tumourous counterparts (Figure 10). Then, we examined the relationship between the expression level of FHL2 and somatic mutations of TP53 gene in the coding region in HCC and non-tumourous adjacent liver samples. TP53 somatic mutations were found in 8 out of 24 tumour samples (Table 1). Most of them were point mutations leading to missense mutations in the central region (303–918) which contains the DNA binding domain. Therefore, it is likely that these mutations affect the DNA binding functions of the p53 protein. Interestingly, the mutation at the codon 249 (nt. 747, G→T) triggering a change in amino acid residue from R to S was found in 2 out of 3 HCC samples with relatively higher FHL2 level. Although the sample size was still too small to draw a definite conclusion, this interesting finding deserves further investigation.

**Table 1:** Correlation between the T/N expression level of FHL2 and coding region mutations of TP53 in HCC samples.

Sample No.	Nucleotidemutation site	Affectcodon (s)	Changes inaa (N-T)	Domain function	FHL2 (T/N)	TP53 (T/N)
312	730 (G→T)	244	G→C	DNA bindingdomain	0.08	0.47
271	376 (T→G)	126	Y→D	DNA bindingdomain	0.10	0.65
348	Δ1008–1010	336–337	ER→D	Oligomerizationdomain	0.10	0.42
426	733 (G→T)	245	G→C	L3 Loop	0.12	1.09
353	644 (G→T)	215	S→I	DNA bindingdomain	0.32	0.41
350	747 (G→T)	249	R→S	L3 Loop	0.94	0.60
447	747 (G→T)	249	R→S	L3 Loop	1.16	0.75
435	1039 (G→A,heterozygous)	347	S→T	Oligomerizationdomain	1.31	1.01

## Discussion

In this study, the activity of FHL2 1a and 1b promoter truncation mutants were determined in HEK 293 cells. Results showed that activities of all FHL2 1a mutants are similar while 1b promoter presented the highest activity at the deletion to −138. The results about the 1b promoter are consistent with the previous finding using Huh7 cells [19]. When comparing the activity between 1a and 1b promoters, we found that the basal activity of the FHL2 1b promoter is much higher than that of 1a in HEK 293 cells. This observation also provides support to the hypothesis that tissue-specific expression pattern of FHL2 might be due to the differential activity of 1a or 1b promoters in different cell types.

Although it was reported that FHL2 is a p53-responsive gene, the precise mechanism of the regulation is still unclear. Here, we show that p53 regulates FHL2 expression at the transcriptional levels. Moreover, according to the results of overexpression and knockdown of p53 in Hep3B and HepG2 liver cells respectively, both the protein and mRNA levels of FHL2 were regulated in a p53-dependent manner. Interestingly, the 1b promoter, which was supposed to be the active form in HEK 293 cells, was insensitive to p53. On the other hand, the 1a promoter showed an active response to p53. Since the 1b promoter is irresponsive to p53, the remarkable increase of the FHL2 protein when TP53 was overexpressed in HEK293 cells was most likely contributed by the activation of 1a promoter. Furthermore, in this study a putative p53 binding site confirmed by the ChIP assay was found in the FHL2 1a promoter. This binding site is located inside the exon 1a, which is not in the upstream region of the transcription start site. Since the elevation of 1a promoter activities by the TP53 overexpression dropped gradually when the promoter became shorter, some other p53 binding sites may also exist. According to the software prediction, three more p53 binding sites were located in position −1871 to −1838, −1013 to −991, +88 to +129. Among them, the sites at −1871 to −1838 and +88 to +129 might be functional binding sites because i) the site at −1871 to −1838 is a common site predicted by 3 prediction programs and the promoter activities suddenly dropped to below 4-fold after deletion to −626; ii) the site at +88 to +129 is commonly identified by all 4 prediction programs. Moreover, it is also within the exon 1a and very close to the validated site at +213 to +232. These two sites may have additional effects on the activation by p53 at the 1a promoter. However, the validated site at +213 to +232 should be the most important site because the mutation in this site can already abolished more than 60% of the promoter activity induced by the TP53 overexpression.

As FHL2 and TP53 were down-regulated in the majority of HCC tumour samples, we further analyse the correlation between the expression levels of TP53 and FHL2-1a. Significant correlation was observed in 41 HCC paired samples. Previous study in our laboratory showed that no mutation in the coding region of FHL2 was found in HCC. In this study, the relationship between the expression level of FHL2 and somatic mutation of TP53 gene in HCC was examined. Our data showed that the frequency of somatic mutations of TP53 was slightly higher in FHL2 down-regulated HCC samples. This result is consistent with the previous report that the expression of FHL2 was decreased in RD cells or osteoblasts expressing mutant TP53 [23]. Nevertheless, a bigger sample size is necessary to confirm this finding. It is also notable that no mutation was found in some of the FHL2 down-regulated tumour samples, implying that somatic mutation of TP53 gene is not the only cause of the decline of FHL2 expression in HCC. Concerning the mutation sites of TP53, it is interesting that the mutation at the codon 249 (nt. 747, G→T) triggering a change in amino acid residue from R to S was found in 2 out of 3 HCC samples with a relatively higher expression of FHL2. R249S mutation is predominantly found in hepatocellular carcinomas in eastern Asia and sub-Saharan Africa. Previous studies had linked it to the exposure to aflatoxin-B1, which is a causative agent of hepatocellular carcinoma [32], [33]. R249S is also a hot spot mutation in the L3 loop of p53 and is structurally essential for stabilizing the hairpin conformation of the L3 loop [34]. Last but not lease, it had been shown to cooperate with HBx mutants to regulate cell proliferation and tumourigenesis in normal hepatocyte cell line [35] and had been associated to large and poorly differentiated HCC [36]. Whether FHL2 expression is altered by this p53 somatic mutant is an interesting topic to be investigated.

In summary, the results of this study suggest a reciprocal transcriptional regulation that might exist between FHL2 and TP53. However, the exact relationship between FHL2 and TP53 in carcinogenesis deserves further investigation.

## Supporting Information

File S1 **Table S1. Clinical features of HCC patients.** The patients were categorized according to their sex, age, tumour size, differentiation status, tumour stage, HBV infection and cirrhosis and fibrosis status. *The total number is less than 41 because of some missing data. **Figure S1. Bioinformatic prediction of p53 binding sites in FHL2 (A) 1a promoter (position** **−2139 to +375) and (B) 1b promoter (position** **−2268 to +397).** Four computational programs were used to predict the p53 binding sites: MatInspector, JASPAR, PROMO and PATCH, representing in red, blue, green, and purple rectangles respectively. The conserved regions among different species are highlighted in blue.
(DOCX)
