# Supplementary material for: Transcriptional Regulation of the Tumor Suppressor FHL2 by p53 in Human Kidney and Liver Cells
Source: PLoS One. 2014 Aug 14;9(8):e99359. doi: 10.1371/journal.pone.0099359 (PMC4133229; doi:10.1371/journal.pone.0099359)

**Supplementary Table 1.** Clinical features of HCC patients. The patients were categorized according to their sex, age, tumour size, differentiation status, tumour stage, HBV infection and cirrhosis and fibrosis status. * The total number is less than 41 because of some missing data.

|  | | *N* (%) |
| --- | --- | --- |
| *Sex* | |  |
|  | Male | 32 (78.0) |
|  | Female | 9 (22.0) |
| *Age (y)* | |  |
|  | <60 | 21 (51.2) |
|  | >=60 | 20 (48.8) |
| *Tumour size (cm)** | |  |
|  | < 5 | 10 (24.4) |
|  | >= 5 | 15 (36.6) |
| *Differentiation** | |  |
|  | Well | 5 (15.6) |
|  | Well to moderate | 5 (15.6) |
|  | Moderate | 16 (50.0) |
|  | Poor | 6 (18.8) |
| *AJCC Staging** | |  |
|  | Stage I | 29 (78.4) |
|  | Stage II | 3 (8.1) |
|  | Stage III | 5 (40.5) |
| *HBV* (HBsAg)* | |  |
|  | Positive | 30 (76.9) |
|  | Negative | 9 (23.1) |

**Supplementary Figure 1**

**Supplementary Figure 1.** Bioinformatic prediction of p53 binding sites in FHL2 **(A)** 1a promoter (position -2139 to +375) and **(B)** 1b promoter (position -2268 to +397). Four computational programs were used to predict the p53 binding sites: MatInspector, JASPAR, PROMO and PATCH, representing in red, blue, green, and purple rectangles respectively. The conserved regions among different species are highlighted in blue.


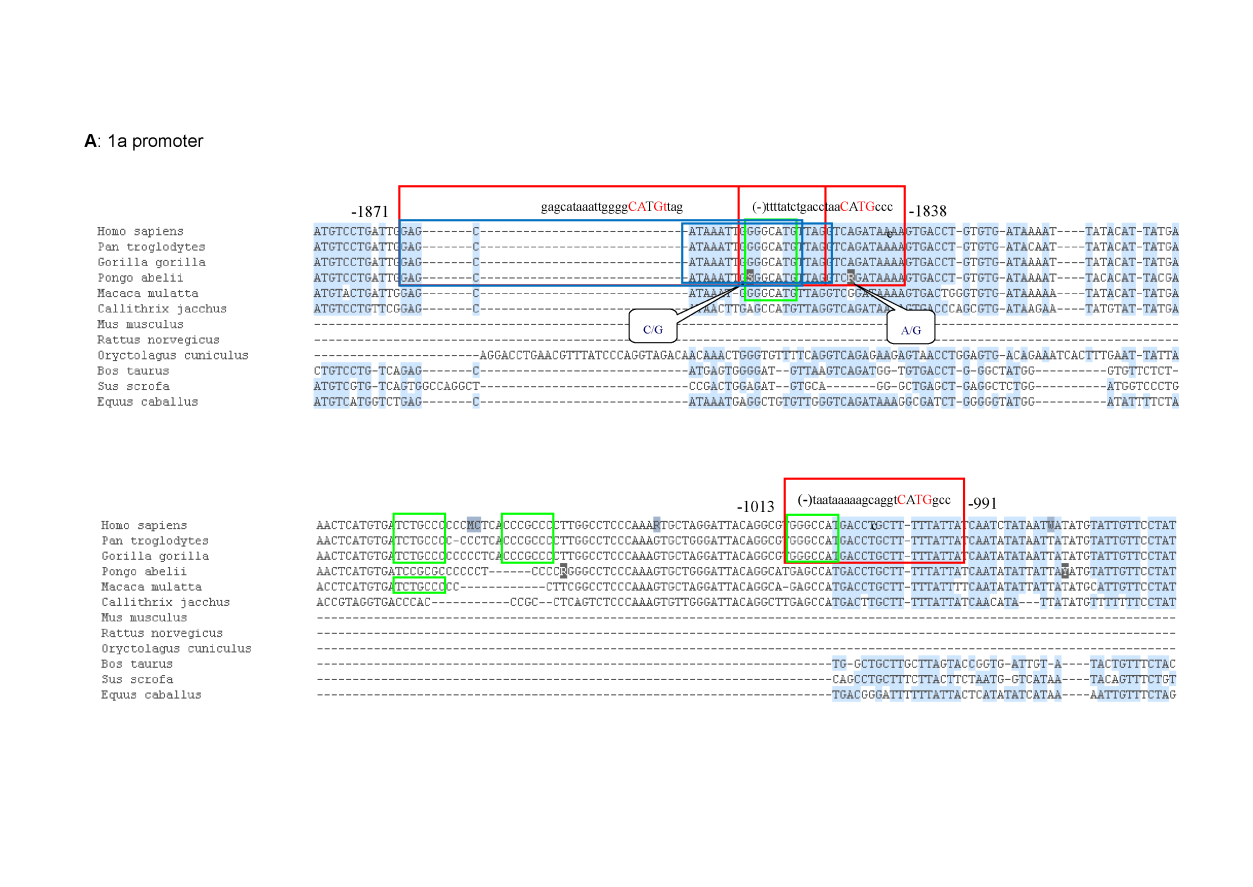


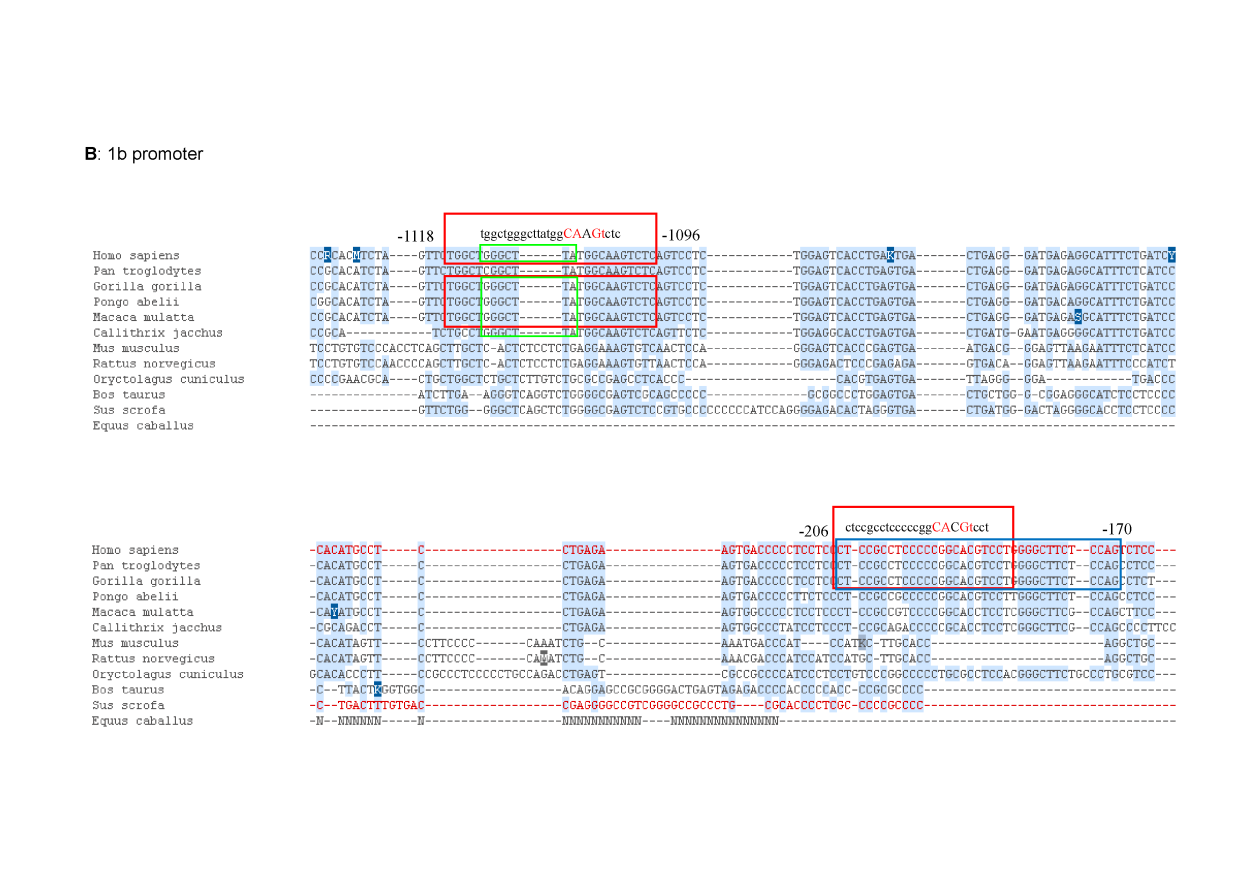


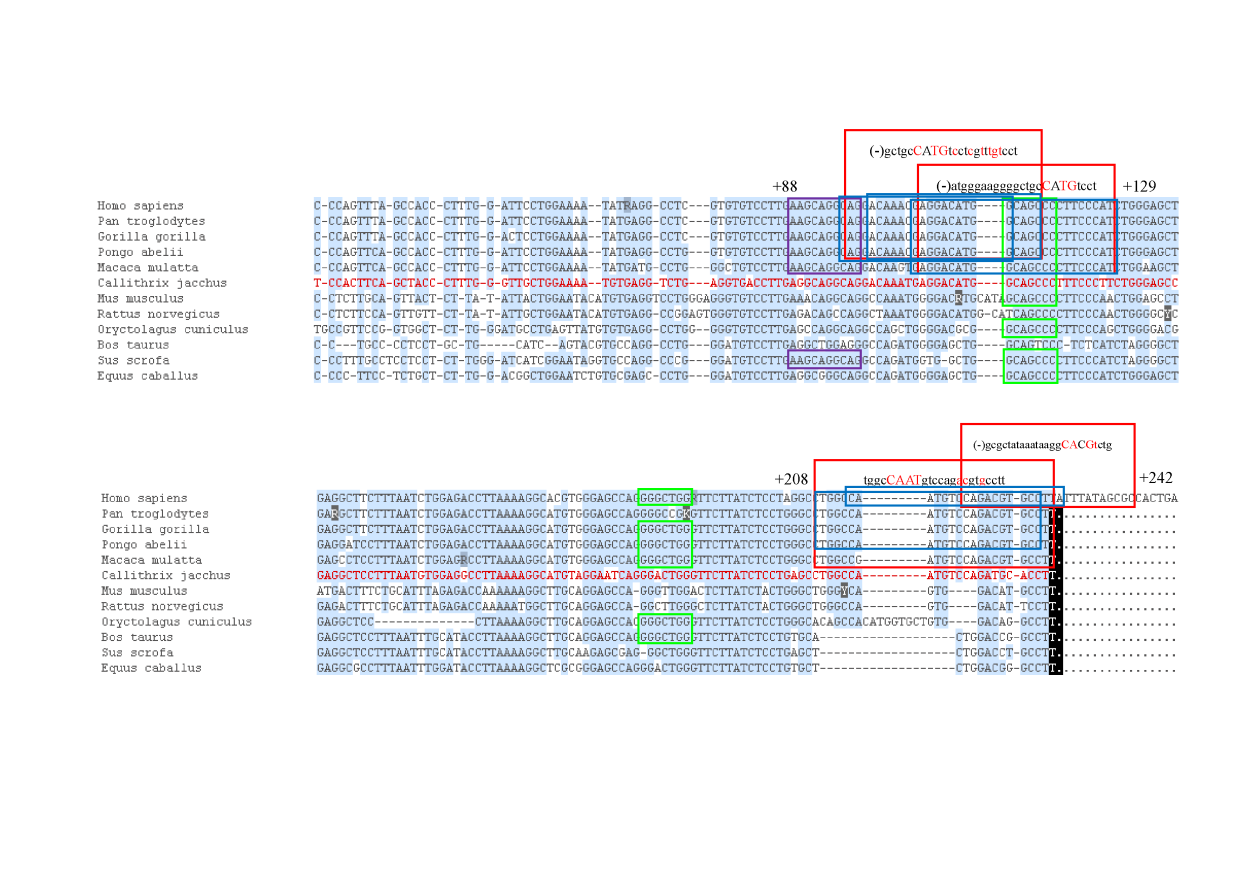

Supplement: File S1 — Table S1. Clinical features of HCC patients. The patients were categorized according to their sex, age, tumour size, differentiation status, tumour stage, HBV infection and cirrhosis and fibrosis status. *The total number is less than 41 because of some missing data. Figure S1. Bioinformatic prediction of p53 binding sites in FHL2 (A) 1a promoter (position −2139 to +375) and (B) 1b promoter (position −2268 to +397). Four computational programs were used to predict the p53 binding sites: MatInspector, JASPAR, PROMO and PATCH, representing in red, blue, green, and purple rectangles respectively. The conserved regions among different species are highlighted in blue. (DOCX) [file pone.0099359.s001.docx]
